# Concise Review: Are Stimulated Somatic Cells Truly Reprogrammed into an ES/iPS-Like Pluripotent State? Better Understanding by Ischemia-Induced Multipotent Stem Cells in a Mouse Model of Cerebral Infarction

**DOI:** 10.1155/2015/630693

**Published:** 2015-04-06

**Authors:** Takayuki Nakagomi, Akiko Nakano-Doi, Aya Narita, Tomohiro Matsuyama

**Affiliations:** Institute for Advanced Medical Sciences, Hyogo College of Medicine, 1-1 Mukogawacho, Nishinomiya, Hyogo 663-8501, Japan

## Abstract

Following the discovery of pluripotent stem (PS) cells such as embryonic stem (ES) and induced pluripotent stem (iPS) cells, there has been a great hope that injured tissues can be repaired by transplantation of ES/iPS-derived various specific types of cells such as neural stem cells (NSCs). Although PS cells can be induced by ectopic expression of Yamanaka's factors, it is known that several stimuli such as ischemia/hypoxia can increase the stemness of somatic cells via reprogramming. This suggests that endogenous somatic cells acquire stemness during natural regenerative processes following injury. In this study, we describe whether somatic cells are converted into pluripotent stem cells by pathological stimuli without ectopic expression of reprogramming factors based on the findings of ischemia-induced multipotent stem cells in a mouse model of cerebral infarction.

## 1. Introduction

Reprogramming by ectopic expression of different transcription factors can induce conversion of adult mammalian somatic cells into various types of stem cells (e.g., induced pluripotent stem (iPS) cells by c-myc, Klf4, Sox2, and Oct3/4 [1, 2]; neural stem cells (NSCs) by c-myc, Klf4, and Sox2 [3]). However, increasing evidence has shown that reprogramming occurs in various organs during natural regenerative processes following injuries [4, 5]. Thus, it may be ideal if endogenous somatic cells can be converted into pluripotent stem (PS) cells under pathological conditions, thereby contributing to regeneration of damaged tissues.

Brain injuries such as ischemia/hypoxia promote the induction of endogenous NSCs [6]. Although the mechanism of NSC induction remains unknown, increasing evidence has shown that ischemia/hypoxia can increase stemness via reprogramming [7, 8]. In support of this idea, we recently showed that brain somatic cells such as pericytes (PCs) within ischemic regions developed stemness, thereby acquiring NSC activity [9–11]. Brain-derived, ischemia-induced stem cells (iSCs) exhibited several PS cell markers such as c-myc, Klf4, Sox2, and Nanog and also showed their multipotency to differentiate into both neural and nonneural cell lineages, presumably through reprogramming [12]. However, it remains unclear whether iSCs can acquire traits similar to those of embryonic stem (ES) and iPS cells.

Recently, Takahashi et al. discovered that somatic cells such as fibroblast cells can be transformed into PS cells with phenotypes similar to ES cells by exogenous expression of Yamanaka's four factors: c-myc, Klf4, Sox2, and Oct4 [1, 2]. Thus, if endogenous somatic cells can be successfully reprogrammed into PS cells in response to stimuli, they must express Yamanaka's four factors. In this study, we described whether stimulated somatic cells can indeed be converted into ES/iPS-like pluripotent state without ectopic expression of reprogramming factors. We focused on the expression of Yamanaka's four factors in iSCs extracted from poststroke adult mouse brains subjected to cerebral infarction.

## 2. What Is the Origin of iSCs?

In the adult mammalian brain, it is well known that NSCs are present in specific brain regions such as the subventricular zone and subgranular zone within the dentate gyrus of the hippocampus and that ongoing neurogenesis is retained in these two zones [13, 14]. Although precise phenotypes of NSCs remain unclear, various types of glial cell lineages such as radial glia [15], astrocytes in the subventricular zone [16], reactive astrocytes [6], resident glia [17, 18], and oligodendrocyte precursor cells [19] have been considered to be possible sources of NSCs.

NSCs are narrowly defined as stem cells that only give rise to neural cell lineages. However, increasing evidence shows that certain NSCs differentiate into both neural and nonneural lineages [20–22]. Thus, in a broad sense, NSCs are defined as multipotent stem cells that can differentiate into various lineages, including neural cells. Although the precise origin, identity, and subtype of such multipotent NSCs remain unclear, we recently demonstrated the development of injury-induced NSCs (iNSCs) within ischemic areas of poststroke brain, with a model of focal cortical infarction in adult mice. These iNSCs possessed self-renewal capacity, which was confirmed by 5-bromo-2′-deoxyuridine uptake. The iNSCs formed neurosphere-like cell clusters* in vitro* and differentiated into electrophysiologically functional neurons, astrocytes, and oligodendrocytes [23]. In addition, we have shown that iNSCs originate, at least in part, from reactive PCs within ischemic regions [9, 10] and that such PCs extracted from ischemic regions (iPCs) exhibited multipotency [12], consistent with the traits of PCs that have multilineage differentiation potential [24–31]. These findings indicate that, under pathological conditions, iPCs may be the origin of iSCs that give rise to iNSCs [9–12].

## 3. Are iSCs ES/iPS-Like Stem Cells?

**Figure 1:**
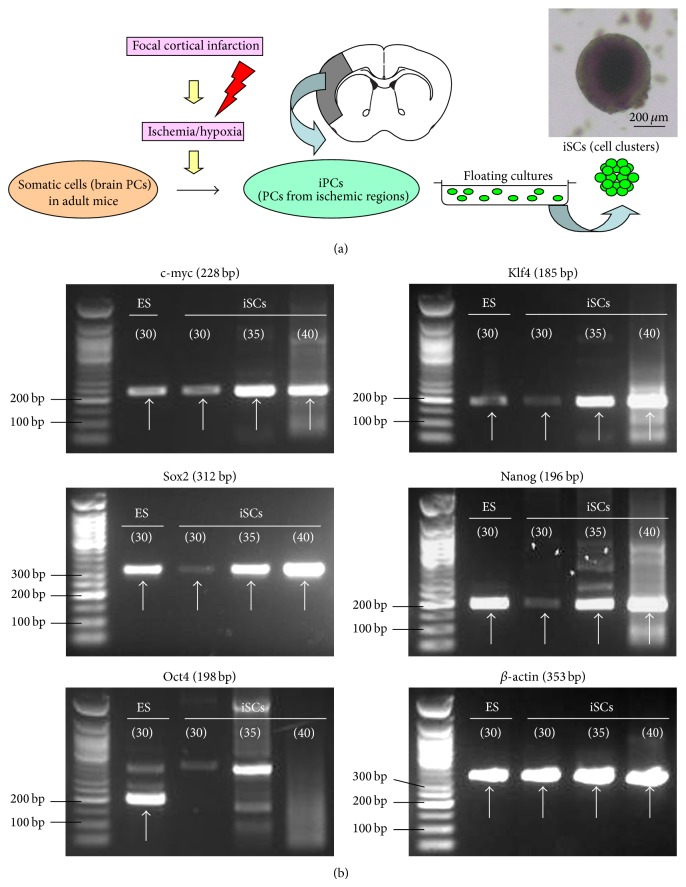
iSCs were isolated from ischemic areas and formed cells clusters (a). iSCs as well as control ES cells expressed c-myc, Klf4, Sox2, and Nanog. However, Oct4 was not observed even after > 35 cycles of PCR amplification. The cycle number of PCR amplification is shown in the circles (b).

**Figure 2:**
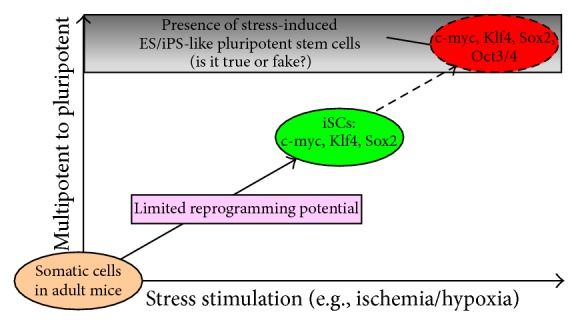
Evidence that iSCs lacking Oct4 have different traits compared with those of ES/iPS cells, suggesting that somatic cells in adult mouse brains have limited reprogramming potential in response to stimuli.

iPC-derived iSCs expressed the NSC marker nestin as well as PC markers such as PDGFR*β*, NG2, and *α*SMA. In addition, iSCs formed cell clusters (Figure 1(a)) and displayed pluripotency markers c-myc, Klf4, and Sox2 of Yamanaka's four factors (Figure 1(b)), as described previously [9, 12], which is consistent with the phenotypes of developing NSCs [32]. Furthermore, iSCs expressed the PS cell marker Nanog, although expression of Nanog as well as Sox2 was weak compared with that of control ES cells. However, our previous study showed that iSCs isolated from adult mouse brain did not express Oct4 by any method, including Western blot [9] and reverse transcriptase-polymerase chain reaction (RT-PCR) analyses [12]. In addition, Oct4 was not observed even after > 35 cycles of PCR amplification (Figure 1(b)), which should be able to detect very low levels of Oct4 in tissue-committed stem cells such as very small embryonic-like stem cells (VSELs) [33]. The evidence that iSCs expressed c-myc, Klf4, and Sox2, but not Oct4, indicates that iSCs have different traits compared with that of PS cells such as ES/iPS cells. This also suggests that somatic cells in adult mouse brain have limited reprogramming potential in response to stimuli, thereby acquiring less stemness compared with that of ES/iPS cells (Figure 2).

One critical question is whether PCs are indeed somatic cells because other groups have suggested that PCs originally possess stemness [24]. However, it is possible that such “naïve” PCs acquire stemness during* in vitro* treatment (e.g., repeated passages and chemical stimulation) because our studies showed that PCs in normal (nonischemic) areas rarely express Yamanaka's factors by immunohistochemistry and Western blot [9, 12]. Furthermore, we have never obtained PCs with stemness from nonischemic areas, which strongly suggests that under normal conditions, PCs in adult mice are initially somatic cells rather than tissue-committed stem cells. Even if PCs originally have a certain degree of stemness, our results still show that it is difficult for PCs in adult mouse brain to become ES/iPS-like stem cells by stimuli alone.

## 4. Why Do iSCs Express Reprogramming Factors following Ischemic Stroke?

iSCs could be easily isolated from ischemic areas but not nonischemic areas [9, 10, 12, 34, 35], showing that ischemia is essential for induction of iSCs. Ischemia is reported to activate the reprogramming factor c-myc following stroke [36]. Consistent with that study, using a mouse model of stroke, we have shown that c-myc^+^ cells were present within ischemic regions, including iSCs; however, they were rarely observed within nonischemic regions [12]. In addition, we showed that the reprogramming factors such as Klf4 and Sox2 were also expressed in iSCs within ischemic regions, whereas they were rarely expressed within nonischemic regions [12]. Furthermore, our study using commercially available primary human pericytes (hPCs) showed that under oxygen-glucose deprivation (OGD) conditions, which mimic ischemia/hypoxia* in vivo* [37], hPCs rapidly increased Klf4 expression [12]. This suggests that OGD is a positive factor for inducing Klf4 following ischemic stroke* in vivo*. However, OGD itself did not affect expression of c-myc in hPCs, although ischemic stroke increased expression of c-myc* in vivo*. In addition, we found that Sox2 expression by OGD treatment was not as remarkable as Klf4 expression, although Sox2 was activated in iSCs following ischemic stroke [12].

These findings indicate that OGD itself cannot completely mimic ischemic events* in vivo* and that factors other than OGD are also involved in the activation of reprogramming factors in iSCs following ischemic stroke. In support of this idea, we found that environmental factors such as leukemia inhibitory factor [38] and basic fibroblast growth factor [39] that are secreted from stimulated endothelial cells surrounding PCs also function as positive factors for activation of Sox2 [12]. These findings indicate that* in vivo* ischemia is a very complex event, and likely multiple factors, including OGD, are involved in the activation of reprogramming factors in iSCs following ischemic stroke. Additional studies are required to clarify which factors/signal pathways are critical to induction of iSCs.

## 5. What Other Previously Reported Stem Cells Express Oct4 Other than ES/iPS Cells?

Although we have never detected Oct4 expression in iSCs, previous reports by other groups have shown Oct4 expression in several types of stem cells other than ES/iPS cells, such as VSELs [33, 40–43] and multilineage-differentiating stress-enduring cells (muse cells) [44, 45]. Ratajczak and colleagues reported that VSELs are a population of developmentally early and small stem cells residing in adult tissues of both mice and humans [40, 42]. VSELs, defined by Lin^−^Sca-1^+^CD45^−^ markers, were reported to have phenotypes of PS cells. VSELs express PS cell markers such as stage-specific embryonic antigen (SSEA)-1, SSEA-4, Nanog, and even Oct4 [42, 46], although Oct4 expression in VSELs was very low compared with that of ES cells [33]. The population of quiescent VSELs expands in response to stimuli [43]. Until now, VSELs were reported to be isolated from various tissues, including bone marrow, testis, and ovary [33, 40, 41]. However, as far as we know, there has been no report of VSELs being obtained from brain. Thus, it is possible that Oct4 expression in stem cells is originally different among organs (e.g., between brains and bone marrow). However, recent studies by several independent researchers cast doubt on the presence of VSELs because they could not find Oct4-expressing PS cells in reported VSEL populations [47–49]. Therefore, the exact characteristics of VSELs should be clarified in further studies.

Muse cells are PS cells which belong to mesenchymal lineages and can be isolated from various tissues (e.g., bone marrows and skin) as SSEA-3/CD105 double-positive cells [44, 45]. In addition, muse cells are reported to express various PS cell markers, including Sox2, Nanog, and Oct4. However, Oct4 expression of muse cells was quite low compared with that of ES cells, and muse cells did not contribute to formation of teratoma-like tumorigenesis. Thus, it seems apparent that muse cells do not have the pluripotency to the degree observed in ES/iPS cells. Until now, muse cells expressing Oct4 can be successfully isolated from humans but not from other species. This also suggests that stemness is originally different among species (e.g., between mice and humans), although their precise traits remain unclear.

There have been many reports in which authors described that various types of stem cells such as mesenchymal stem cells [50, 51], adipose-derived stromal cells [52], and neural crest-derived stem cells [53–55] expressed Oct4. However, Bhartiya implied that some studies may have misinterpreted Oct4 expression [33]. Takeda et al. showed that Oct4 has two major isoforms, Oct4A and Oct4B, and only Oct4A is related to pluripotency, whereas Oct4B has no biological function [56]. Further, Warthemann et al. pointed out that it is possible that a false-positive for Oct4A expression may lead to misinterpretation [57]. Thus, stem cell biologists should carefully investigate Oct4 expression using appropriate positive controls such as ES/iPS cells because Oct4 is a key factor when estimating the state of pluripotency [1, 2, 58].

## 6. Conclusion

Herein, we discussed in detail whether stimulated somatic cells can acquire pluripotency. Our studies regarding iSCs showed they expressed c-myc, Klf4, and Sox2, but not Oct4, suggesting that even with severe stimuli, such as ischemia/hypoxia, which promote reprogramming, it is not easy to reprogram adult somatic cells into an ES/iPS-like state. Although iSCs lacked Oct4, pluripotency of iSCs should be carefully investigated because they certainly express various pluripotent markers such as c-myc, Klf4, Sox2, and Nanog, as we previously demonstrated [9, 12]. We need further examination regarding teratoma formation capacity and germline transmission ability. We also understand that further studies are required using younger mice (e.g., neonatal mice), various organs (e.g., bone marrow, liver, and lung), various stimuli (e.g., sheer stress), and/or other species (e.g., rat and human). We hope that stem cell biologists would provide convincing answers for these fundamental questions in future investigations.

## Abbreviations

ES cells: Embryonic stem cells
iNSCs: Ischemia-induced neural stem cells
iPCs: Pericytes extracted from ischemic regions
iPS cells: Induced pluripotent stem cells
iSCs: Ischemia-induced stem cells
NSCs: Neural stem cells
PCs: Pericytes
PS cells: Pluripotent stem cells
hPCs: Human pericytes
VSELs: Very small embryonic-like stem cells
SSEA: Stage-specific embryonic antigen
RT-PCR: Reverse transcriptase-polymerase chain reaction
OGD: Oxygen-glucose deprivation.
